# TLR2 deficiency promotes IgE and inhibits IgG1 class-switching following ovalbumin sensitization

**DOI:** 10.1186/s13052-021-01088-3

**Published:** 2021-07-27

**Authors:** Yuqin Li, Qiu Chen, Wei Ji, Yujie Fan, Li Huang, Chu Chu, Weifang Zhou

**Affiliations:** 1 Children’s Hospital of Soochow University, Suzhou, 215003 People’s Republic of China; 2 School of Radiation Medicine and Protection, Soochow University, Suzhou, 215123 China

**Keywords:** Ovalbumin, Sensitization, TLR2, IL21, STAT3, IgG1, IgE

## Abstract

**Background:**

To explore the roles of Toll-like receptor (TLR)2 in Th2 cytokine production and immunoglobulin (Ig) class switching following ovalbumin (OVA) sensitization.

**Methods:**

TLR2^−/−^ and wild-type C57BL/6 mice were sensitized by intraperitoneal injection with OVA. Lung pathology was assessed by hematoxylin and eosin staining. Abundance of interleukin (IL)4, IL5, IL13, and IL21 transcripts in the lungs was quantified by RT-PCR. OVA-specific IgG1, IgG2a, IgG2b, IgE and IgM were quantified by enzyme-linked immunosorbent assay. Phosphorylated signal transducer and activator of transcription (STAT)3 in lung tissue was detected by immunohistochemistry staining and nuclear factor (NF) κB activation was measured by immunofluorescence staining. STAT3 activation was inhibited using cryptotanshinone (CPT) treatment. Germline transcripts (Iμ-Cμ, Iγ-Cγ, Iα-Cα or Iε-Cε), post-recombination transcripts (Iμ-Cγ, Iμ-Cα or Iμ- Cε) and mature transcripts (V_H_DJ_H_-Cγ, V_H_DJ_H_-Cα or V_H_DJ_H_-Cε) were analyzed from splenic B cells of OVA-sensitized wild-type mice (with or without CPT treatment) and TLR2^−/−^ mice (with or without IL21 treatment).

**Results:**

The lungs of TLR2^−/−^ mice showed a lesser degree of inflammation than wild-type mice after OVA sensitization. Following OVA sensitization, levels of IL4, IL13, and IL21, but not IL5, were significantly lower in TLR2^−/−^ compared with wild-type mice. Moreover, OVA-specific IgG1 and IgE titers were markedly lower and higher, respectively, in TLR2^−/−^ mice. TLR2 deficiency inhibited STAT3 activation but not NF-κB p65 activation. CPT treatment reduced IgG1 titers via inhibition of Stat3 phosphorylation. Both TLR2 knockout and CPT treatment reduced the frequencies of Iγ1-Cγ1, Iγ3-Cγ3 and Iα-Cα transcripts, but IL21 treatment compensated for the effects of TLR2 deficiency.

**Conclusion:**

These results suggest a role of TLR2 in restricting OVA-sensitized lung inflammation via promotion of IgG1 and inhibition of IgE class switching regulated by IL21 and STAT3.

## Background

Asthma and allergic diseases have become major public health concerns of the twenty-first century in developed countries [1]. Two phases are involved in the development of allergic disease: sensitization and elicitation. The effects of sensitization are critical but delayed, and allergic diseases could be cured if sensitization can be avoided.

A key event in the pathogenesis of sensitization is the production of IgE antibodies. IgE plays a central role in allergic immune responses and is essential for host defense against pathogens in mucosal tissues [2]. Other critical immunoglobulin (Ig) isotypes exist in serum and mucosal secretions, including IgA, IgM and IgG [3]. Normally, serum levels of IgE are very low compared with IgG, but elevated levels of IgE are observed in patients with allergic diseases [4]. Reducing IgE concentrations alters allergic immune responses such as those observed in asthma and hyper-IgE disorder [5]. However, bronchial inflammation in response to allergen inhalation can occur in the presence or absence of IgE [6]. Moreover, IgE is not required for the induction of marked allergic airways inflammation in response to inhaled allergens in mouse models. Allergic diseases are typically characterized by a type 2-biased inflammation. Accumulating evidence indicates that antigen-specific Th2 cells and their cytokines such as IL-4, IL-5, and IL-13 orchestrate these pathognomonic features of allergic diseases. Administration of IL15, but not allergen-specific IgE, to sensitized, IL4–deficient mice prior to allergen airway challenge restored eosinophilic airway inflammation [7]. It is well established that certain cytokines regulate Ig isotype switching in vitro and in vivo and that these same cytokines also stimulate germline Ig C_H_ gene transcription [8]. In the mouse, IL4 stimulates germline γ1 and ε-Ig gene transcription [9–11]. IL4 and IL13 are closely related cytokines that are produced by Th2 cells. IL4^−/−^ and IL4/IL13^−/−^ mice produced almost no IgE and were highly resistant to OVA-induced diarrhea, whereas allergic diarrhea was only partially impaired in IL13^−/−^ and IL13Ralpha1^−/−^ mice [12]. IL-21 is a pleiotropic cytokine that can influence the activation, differentiation, and expansion of B and T cell effector subsets. IL21 has a significant influence on regulation of B cell function in vivo and cooperates with IL4 [13]. IL21 has been recognized to suppress IL4-induced IgE production by murine B cells. IL21R deficiency was associated with increases in phosphate-buffered saline(PBS)- and allergen-driven IgE levels, while IgG1 and IgG2a levels were decreased [14]. Surface antibody-positive B cells, at this stage, remain sensitized to the allergen.

Nuclear transcription factors play important roles in sensitization and antibody production. Both IL4 and IL13 activate signal transducer and activator of transcription factor 6 (STAT6) [15]. Wan et al. reported that IL21-mediated induction of STAT1 phosphorylation was higher in CD4^+^ T cells from patients with autosomal dominant hyper-IgE syndrome (which is caused by STAT3 deficiency) as well as in cells from patients with STAT1 gain-of-function mutations [16].

Toll-like receptors (TLRs) play critical roles in directing the course of acquired immune responses. B cell-restricted MyD88-knockout mice showed reduced IgE/IgG1 production in response to lung inhalation of ovalbumin (OVA) [17]. TLR2-specific ligands (except peptidoglycan) augmented secretion of histamine or leukotriene C4 in response to IgE-dependent activation and secretion of IL13 in response to IgE-independent stimulation [18]. Class switch recombination (CSR) to specific Ig isotypes requires NF-κB transcription factors in B cells. NF-κB composition in B cells is also highly regulated and can vary significantly depending upon how B cells are activated [19].

Our previous report [20] suggested that TLR2 knockout in mice alleviated asthma. Levels of p38/AKT/ NF-κB p65 and phosphorylated (p) extracellular signal-regulated kinase (ERK) were decreased in the lungs of OVA-sensitized and -challenged TLR2^−/−^ mice. Redecke et al. [21] reported that activation of TLR2 before sensitization heightened experimental asthma. However, TLR2 activation immediately prior to intranasal challenge reduced allergic airway inflammation [22–25]. More work is required to understand the varied roles of TLR2 during allergic sensitization and challenge. In this study, we examined the roles of cytokine expression, Ig class switching and nuclear transcription factors in OVA sensitization of TLR2^−/−^ mice, with the goal of understanding the role of TLR2 in OVA sensitization in detail.

## Methods

### Mice, treatments and B-cell sorting

Six-to-eight-week-old male C57BL/6 (SLACCAS, Shanghai, China) and TLR2^−/−^ (B6.129-Tlr2^tmIkirNJU^, Model Animal Research Center of Nanjing University, Jiangsu, China) mice were bred and housed in a pathogen-free facility. The temperature and humidity were 18–22 °C and 30–50%, respectively, and a 12- h day and night cycle was maintained. These mice were provided with laboratory water and food ad libitum. All applicable international, national, and/or institutional guidelines for the care and use of animals were followed. The mice were sensitized by intraperitoneal injection of 100 μg of OVA (Sigma, St. Louis, MO, USA) a 400 μg Al(OH)_3_ (Sigma, St. Louis, MO, USA) in a volume of 0.5 mL on days 0 and 7. Control mice were treated in an identical manner except with saline replacing OVA. Cryptotanshinone (CPT)-treated mice received intraperitoneal injections of CPT (Selleck, Houston, TX, USA) dissolved in 0.5 mL of 0.9% saline solution. CPT was administered at doses of 200 mg/kg/day on days 0 and 7, 30 min prior to each OVA sensitization. Either 1 or 2 μg of mIL21 (Peprotech) was dissolved in 0.9% saline solution and administered to selected TLR2^−/−^ mice (25 g/mouse, *n* = 6 for each dose) prior to each OVA sensitization.

Mice were euthanized by CO_2_ asphyxiation. The mice were placed in a box and 100% carbon dioxide was introduced. The filling rate is about 20% of the volume of the chamber per minute. Spleens were harvested using standard dissection techniques and placed in wells of tissue culture–grade six-well plates containing 2 mL of cold RPMI medium on ice. Splenocytes were isolated by physically dissociating spleens between the frosted ends of two sterile glass slides. Cells were washed off the glass slides by pipetting 1–2 mL of RPMI medium onto the slides. The cells were filtered through a 40-μm cell strainer and centrifuged at 430×g at 4 °C for 5 min to pellet cells. The supernatant was removed and the cells were incubated with red blood cell lysis buffer (BD, 0.5 mL per spleen) on ice for 5 min. The lysis reaction was stopped by addition of cold PBS. Settled cell debris was removed with a pipette and the supernatant containing splenocytes was transferred to a new tube, taking care to avoid transfer of cell debris. The tube was centrifuged at 430×g at 4 °C for 5 min and resuspended in PBS containing 1% (v/v) fetal bovine serum. Cells were counted using a hemocytometer and normalized to 10^8^ cells/mL. Each cell sample was incubated with the following antibodies at 4 °C for 25 min: CD45-PE, CD4-FITC, and CD45R/B220-APC (all from eBioscience). After washing, CD45^+^CD4^−^CD45R/B220^+^ cells were sorted and collected (BD) for RNA extraction.

### Real-time polymerase chain reaction (RT-PCR)

Lung tissue or B-cell RNA was extracted using Trizol (Invitrogen, Carlsbad, CA) following the manufacturer’s protocol. Briefly, 1 μg of total RNA was used for reverse transcription (Takara RNA PCR kit, Shiga, Japan) according to the manufacturer’s protocol. The resulting cDNA was analyzed and amplified using RT-PCR (ABI PRISM7500, USA). The 20-μL reactions consisted of 2 μL of cDNA, 10 μL of SYBR Green I Master (Roche, Mannheim, Germany), and 1 μL each of forward reverse primers. The reactions were incubated at 95 °C for 10 min and then subjected to 30 cycles 95 °C for 15 s, 60 °C for 15 s, and 72 °C for 30 s. All samples were analyzed in triplicate. Transcripts were quantitated by their comparative threshold cycle number (Ct). The sequences of all primers are shown in Table 1. β-actin served as an internal control. We chose the comparative Ct (ΔCt) quantification method to quantitate transcript abundance. CSR germline transcripts, post-recombination transcripts and mature transcripts were examined by RT-PCR using previously-described primers (Pone et al. 2012).

**Table 1:** Primer listed for real-time PCR

RNA	Forward Primer	Reverse Primer
β-actin	GACGGGGTCACCCACACTGT	AGGAGCAATGATCTTGATCTTC
IL-4	GGTCTCAACCCCCAGCTAGT	GCCGATGATCTCTCTCAAGTGAT
IL-5	CTCTGTTGACAAGCAATGAGACG	TCTTCAGTAT GTCTAGCCCCTG
IL-13	CCTGGCTCTTGCTTGCCTT	GGTCTTGTGTGATGTTGCTCA
IL-21	ATGCCCTTCCTGTGATTCGT	TCTGTGGGAACGAGAGCCTA

### Lung histology, immunofluorescence and immunohistochemistry

Mouse lung tissue was soaked in 10% neutral formalin until lobes separated, then embedded in paraffin, cut into 3-μm thick sections, and stained with hematoxylin and eosin.

Tissue sections were deparaffinized, rehydrated, and subjected to antigen retrieval by heating. The sections were incubated in 3% (v/v) H_2_O_2_ for 10 min and then in bovine serum albumin solution for 1 h. Primary antibodies against NF-κB p65 (1:200, Santa Cruz biotechnology, San Francisco, USA) or p-STAT3 (Cell Signaling Technology, Danvers, MA, USA) was then added overnight at 4 °C. On the following day, secondary antibodies (PE-conjugated donkey anti-goat IgG, 1:1000, Beyotime, Shanghai, China or goat anti-mouse/rabbit IgG, GK5005, Gene Tech, Shanghai, China) were added to the slides for 0.5–1 h at room temperature. DAPI was added to the slides for 10 min. Tissue sections incubated with STAT3 were stained for 40 s with hematoxylin and then dehydrated and sealed. The samples were observed and photographed using an Olympus microscope.

### Serum OVA-specific Ig and Ig subclass measurement by ELISA

Blood samples were allowed to clot for at least 60 min and then centrifuged at 400×g for 10 min. Serum was thus obtained for analyses of IgG1, IgG2a, IgG2b, IgA, IgE and IgM. Serum Ig content were estimated using solid-phase indirect ELISA kits (Chondrex, Redmond, WA, USA) according to the manufacturer’s instructions. Each standard and sample were tested three times. Absorbance was measured at 450 nm using an ELISA plate reader (Labsystems, MultiskanEX, Finland). The results were expressed in grams per liter.

### Statistical analysis

All data were presented as means ± standard deviations. Analysis of multiple comparisons was carried out by ANOVA. T-tests were used to assess differences between groups. Statistical significance was assumed for *P* < 0.05. All statistical analyses were performed using SPSS 17.0 software.

## Results

### TLR2 knockout reduced lung inflammation induced by OVA sensitization

**Fig. 1:**
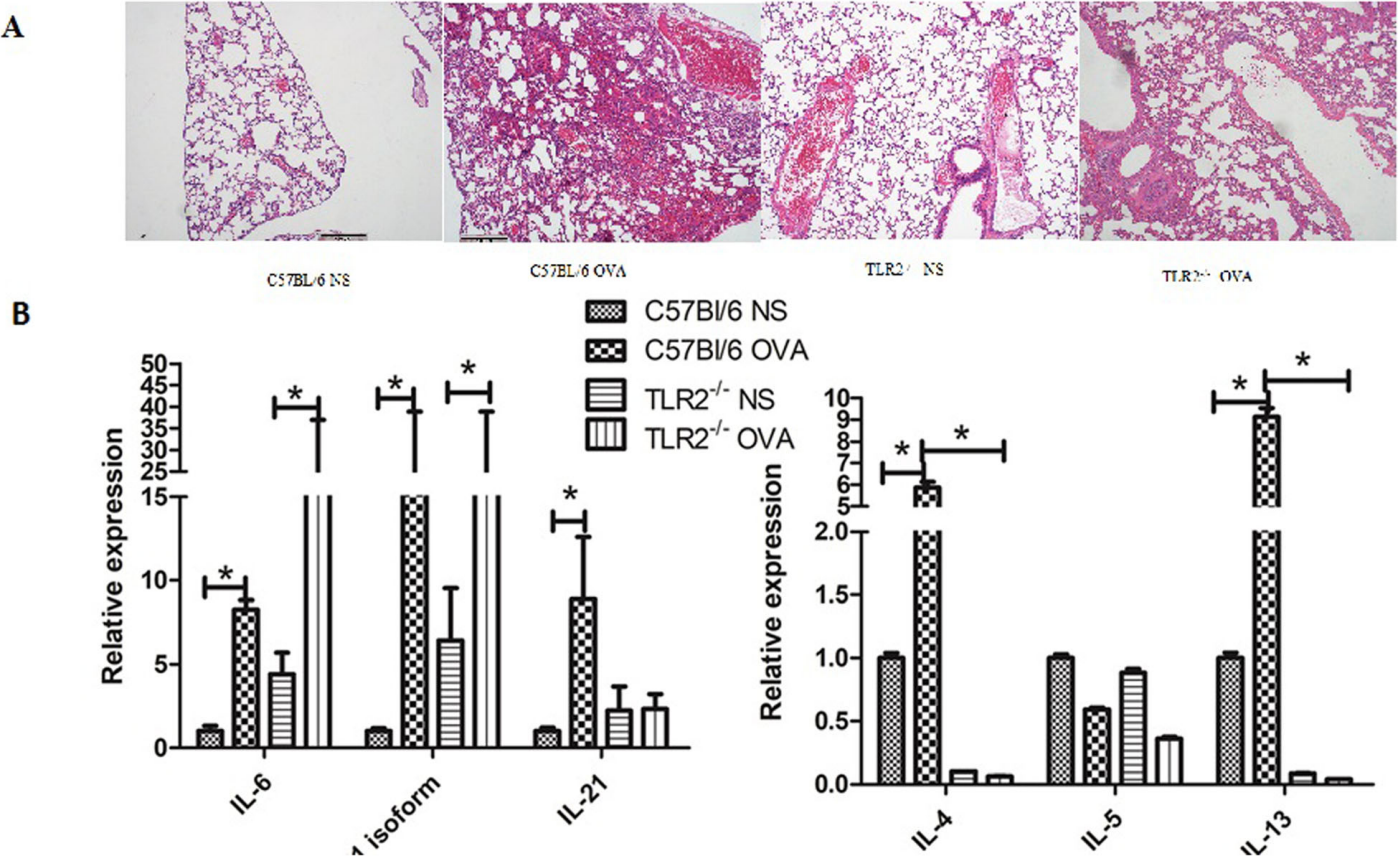
The lungs of TLR2^−/−^ mice showed less inflammation following OVA sensitization compared with wild-type mice. **A** Lungs were fixed, embedded in paraffin and cut into 3-μm thick sections. Lung sections were stained with hematoxylin and eosin (HE) and photographed using an Olympus microscope. Magnification: 200-fold; **B** Cytokines were measured in Lung tissue or B-cell by RT-PCR. Data are shown individually and as the mean ± s.e.m. IL4, IL13 and IL21 expression decreased more significantly following OVA sensitization in TLR2^−/−^ mice compared with wild-type mice as shown. Data for IL4, IL5, IL13 and IL21 were normalized to wild-type mice treated with normal saline (NS). *: *p* < 0.05, *n* = 6 mice per group

As shown in Fig. 1, the lungs of TLR2^−/−^ mice showed less inflammatory cell infiltration following OVA sensitization than those of wild-type mice (Fig. 1a). Levels of IL4 and IL13 induced by OVA sensitization were significantly lower in TLR2^−/−^ mice (Fig. 1b). IL6 and IL21 were upregulated after OVA sensitization in both wild-type and TLR2^−/−^ mice. However, TLR2^−/−^ mice showed reduced IL21 upregulation in response to OVA sensitization. There was no significant difference in IL5 expression between wild-type and TLR2^−/−^ mice irrespective of OVA treatment.

### TLR2^−/−^ mice had decreased serum titers of OVA-specific IgG_1_ but increased titers of OVA-specific IgE following OVA sensitization

**Fig. 2:**
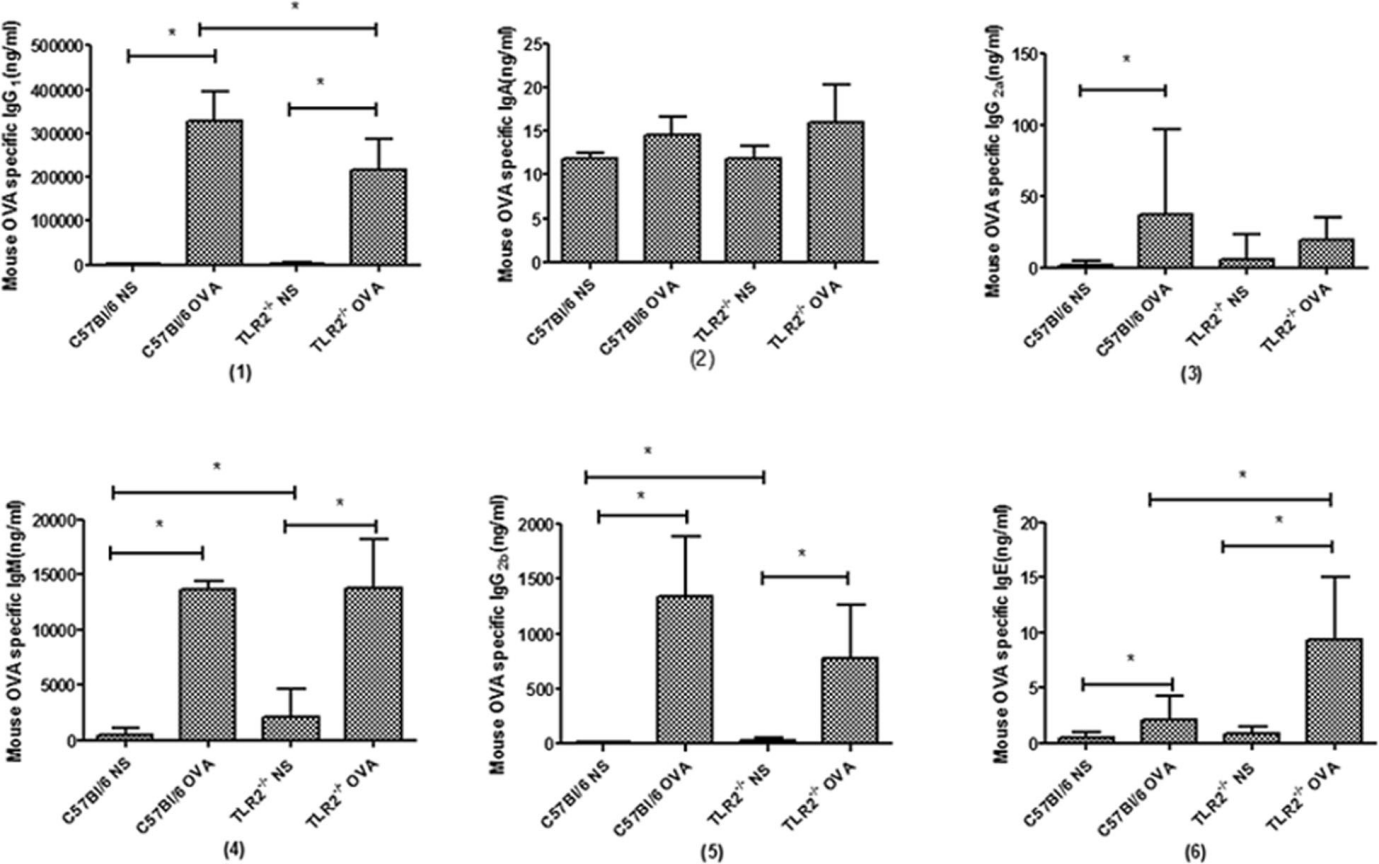
Serum titers of OVA-specific IgG_1_(1), IgA (2), IgG_2a_ (3), IgM (4), IgG_2b_ (5) and IgE (6) in wild-type and TLR2^−/−^ mice were detected by ELISA. Data are shown individually and as the mean ± s.e.m. OVA-specific IgG1 and IgE titers were markedly lower and higher, respectively, in TLR2^−/−^ mice. *: *p* < 0.05, *n* = 6 mice per group

Wild-type mice developed serum titers of OVA-specific IgG_1_ (Fig. 2(1)), IgG_2a_ (Fig. 2(3)), IgG_2b_ (Fig. 2(5)), IgM (Fig. 2(4)) and IgE (Fig. 2(6)) after OVA sensitization. The titers were highest for IgG_1_ and IgM. Titers of OVA-specific IgG_1_ were 140,000 times higher than OVA-specific IgE. TLR2^−/−^ mice developed serum titers of OVA-specific IgG_1_, IgG_2b_, IgM and IgE after OVA sensitization. Compared with OVA-sensitized wild-type mice, OVA-sensitized TLR2^−/−^ mice had decreased serum titers of OVA-specific IgG_1_ (> 300,000 ng/mL vs 200,000 ng/mL) but increased serum titers of OVA-specific IgE (2 vs 9 ng/mL).

### NF-κB was not activated by OVA sensitization in either wild-type or TLR2^−/−^ mice

**Fig. 3:**
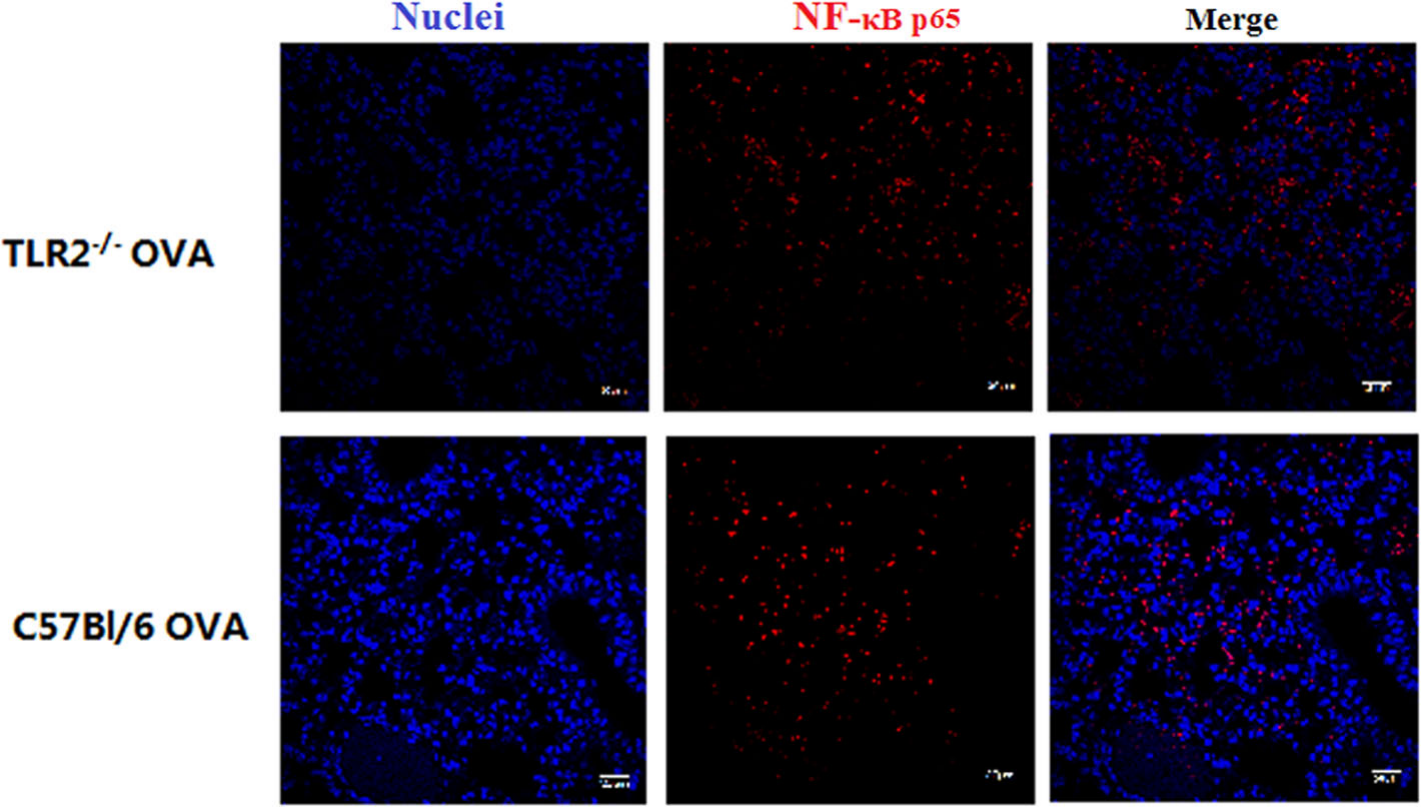
Immunofluorescence staining of NF-κB p65 (PE, red) showed that p65 was not detectable in nuclei (DAPI, blue). Bar = 30 μm

As shown in Fig. 3, NF-κB p65 (red) was located outside of nuclei (blue) in the lungs of wild-type mice; thus, NF-κB was not activated and did not translocate to nuclei following OVA sensitization. As expected, similar results were obtained in TLR2^−/−^ mice. Thus, the lower lung inflammation observed in TLR2^−/−^ mice following OVA sensitization compared was not due to differences in NF-κB activation.

### TLR2^−/−^ mice showed reduced STAT3 phosphorylation in the lung following OVA sensitization (Fig. 4)

**Fig. 4:**
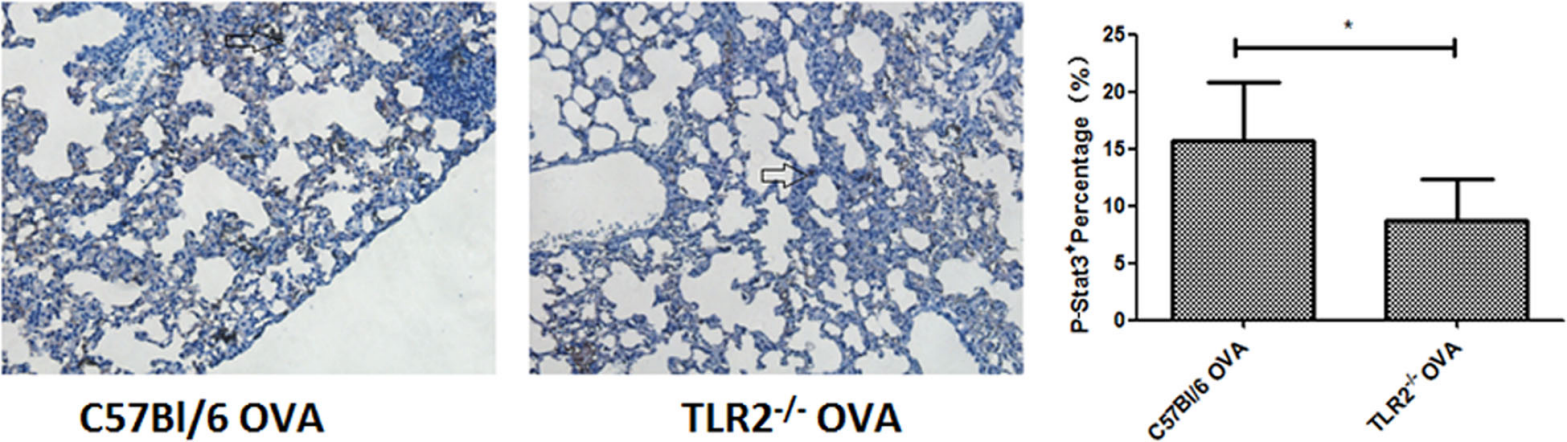
Immunohistochemistry staining of p-STAT3 showed lower numbers of positive epithelial cells in the lungs of OVA-sensitized TLR2^−/−^ mice compared with those of OVA-sensitized wild-type mice. Magnification: 200-fold. *: *p* < 0.05, *n* = 6 mice per group

In the lungs of OVA-sensitized wild-type mice, 15% of epithelial cells stained positive for p-STAT3, while in the lungs of OVA-sensitized TLR2^−/−^ mice, only 9.8% of epithelial cells of were p-STAT3-positive. Compared with OVA-sensitized wild-type mice, the number of p-STAT3-positive cells in the lungs of OVA-sensitized TLR2^−/−^ mice was significantly lower.

### CPT inhibited p-STAT3 expression in lung epithelial cells of OVA-sensitized wild-type mice, decreased serum titers of OVA-specific IgG_1_, and alleviated lung inflammation (Fig. 5)

**Fig. 5:**
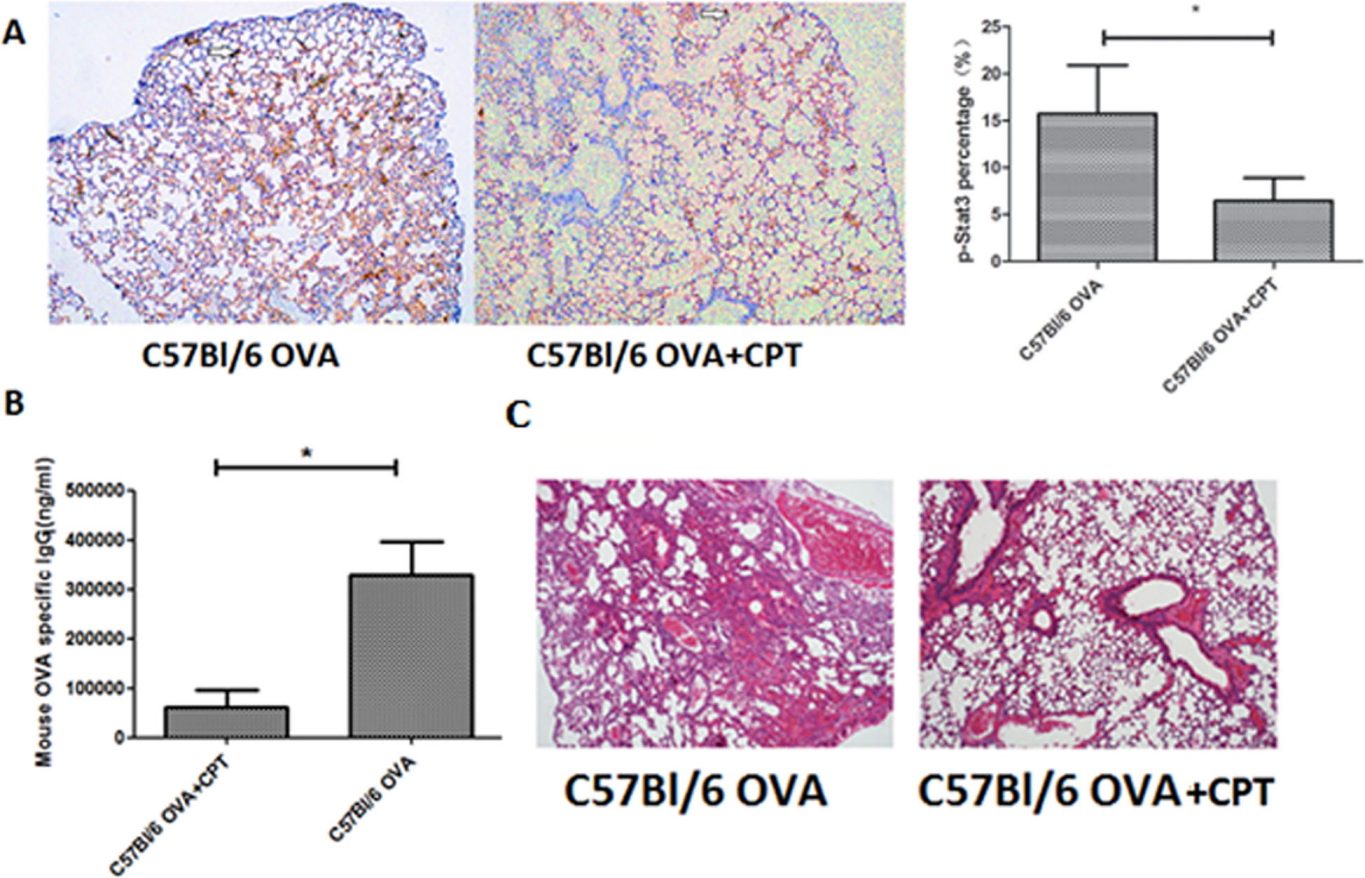
CPT administered at doses of 200 mg/kg/day on days 0 and 7 30 min prior to OVA administration decreased levels of p-STAT3 in lung epithelial cells of wild-type mice (**A**, immunohistochemistry), decreased serum titers of OVA-specific IgG_1_ (**B**, ELISA) and alleviated lung inflammation (**C**, hematoxylin and eosin staining). A and C magnification: 200-fold. *: *p* < 0.05, *n* = 6 mice per group

Treatment of wild-type mice with the STAT3 inhibitor CPT3 resulted in decreased frequency of p-STAT3-positive lung epithelial cells (from 15 to 6%) and also a 75% decrease in OVA-specific IgG_1_ (from 320 μg/mL to approximately 80 μg/mL). CPT treatment greatly reduced lung inflammation and congestion.

### CPT-treated splenic B-cells from wild-type mice and splenic B-cells from TLR2^−/−^ mice had lower levels of germline transcription that were reversed by addition of IL21 (Fig. 6)

**Fig. 6:**
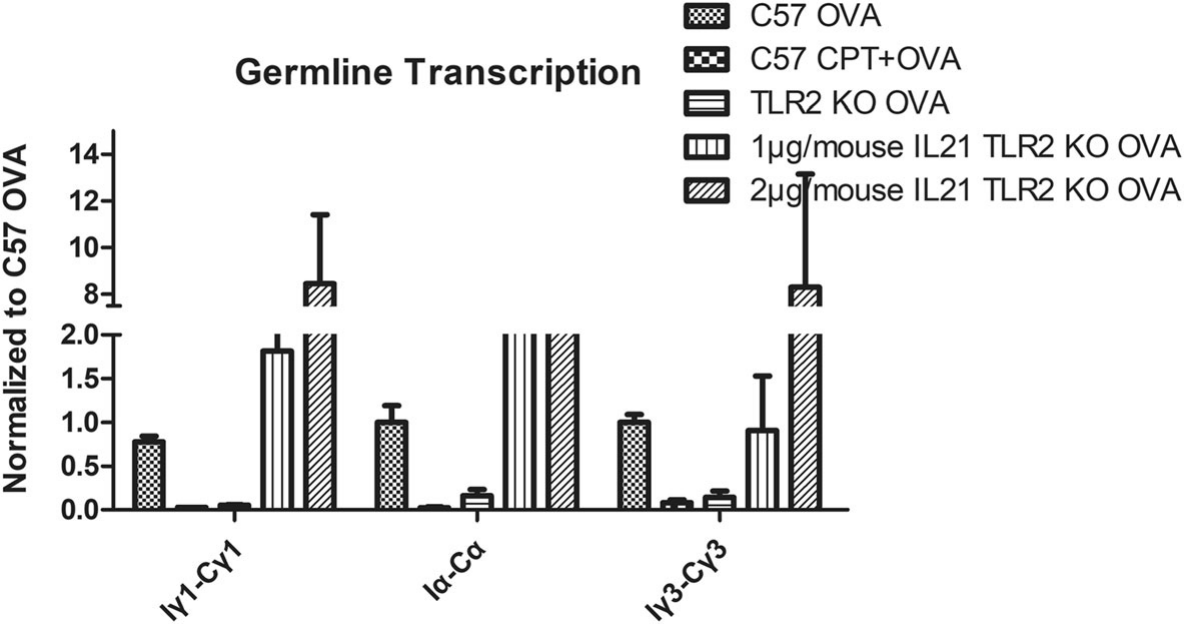
RT-PCR showed that addition of IL21 addition Iα-Cα, Iγ1-Cγ1, and Iγ3-Cγ3 fgermline transcription in CPT-treated wild-type or TLR2^−/−^ mice, *n* = 6 mice per group, *, *p* < 0.05

Germline transcripts (Iμ-Cμ, Iα-Cα, Iε-Cε, Iγ1-Cγ1, Iγ2a-Cγ2a or Iγ3-Cγ3), post-recombination transcripts (Iμ-Cγ, Iμ-Cα or Iμ-Cε) and mature transcripts (V_H_DJ_H_-Cγ, V_H_DJ_H_-Cα or V_H_DJ_H_-Cε) were analyzed. Both CPT treatment of wild-type mice and TLR2 knockout nearly abrogated germline Iα-Cα, Iγ1-Cγ1, Iγ3-Cγ3 transcripts in splenic B cells. Administration of 1 μg of IL21 per mouse restored Iα-Cα, Iγ1-Cγ1, and Iγ3-Cγ3 expression and 2 μg of IL21 promoted higher levels of expression. However, Iε-Cε transcripts could not be detected in either group.

## Discussion

Our results showed that in wild-type mice, airway infiltration by inflammatory cells following OVA sensitization was obvious, and mainly included lymphocytes and eosinophils. Compared with mice treated with NS, OVA-sensitized mice showed a decreased proportion of macrophages in alveolar lavage fluid and increased numbers of eosinophils. Following OVA sensitization, expression of Th2 cytokines (IL4 and IL13) was significantly upregulated, while there was no significant difference in IL5 expression. Serum titers of OVA-specific IgG1, IgA, IgG2a, IgG2b, IgM and IgE increased significantly in wild-type mice following OVA sensitization. These results indicated that OVA sensitization promotes an inflammatory response.

IL4, IL13 and IL21 expression was almost undetectable in TLR2^−/−^ mice irrespective of OVA treatment. Serum titers of OVA-specific IgE and IgG1 were increased in TLR2^−/−^ mice after stimulation with OVA compared with NS. However, OVA-sensitized TLR2^−/−^ mice had higher serum titers of OVA-specific IgE but lower titers of OVA-specific IgG1 compared with OVA-sensitized wild-type mice. IL21 isoform showed a different expression pattern than IL21 and was upregulated to a greater extend in TLR2^−/−^ mice sensitized with OVA compared with wild-type mice. Nara et al. reported that IL21 isoform had similar functions as IL21: it could promote phosphorylation of STAT3 but was seldom secreted from the cells, and thus had primarily autocrine function [26]. IL21 is a product of Th17 cells [27], which co-localize with B cells both in spleen or lymph node [28], and so it was suggested that only IL21 and not IL21 isoform acted on B cells. Liu et al. [29] examined the effect of combining IL21 with TLR7/8 or 9 agonists and found that IL21 could increase the activity of the TLR–MyD88–STAT3 pathway in human B cells by enhancing phosphorylation of STAT3 and promoting IgG production. TLR2 had similar synergy with TLR7/8 or 9 in promoting IgG1 germline transcription and secretion from mouse splenic B cells. These findings suggested that downregulation of IL21 in TLR2^−/−^ mice may be responsible for the higher serum titers of OVA-specific IgE and lower serum titers of OVA-specific IgG1 in OVA-sensitized TLR2^−/−^ mice [13].

IgE plays a central role in the early asthmatic response and can bind to allergens, causing degranulation of mast cells or basophils and triggering inflammation. Inflammatory mediators cause mucosal edema, smooth muscle contraction, and increased glandular secretions to initiate an allergic inflammatory response. Anti-IgE therapy impairs its action by reducing the amount of free IgE available to bind to effector cells [30]. However, treatment responses vary widely among individuals [31]. Besides IgE, the antibody isotype that gives rise to sensitization and allergic asthma, the immune response to common inhaled allergens also includes IgG. Bronchoalveolar lavage fluid of OVA/OVA mice contained 150 mg/mL of OVA-specific IgG and 8.93 mg/mL OVA-specific IgE [32]. In addition, it has been suggested that IgG1 is closely related to Th2 responses [33–36]. Thus, in our study, the lower OVA-induced lung inflammation of TLR2^−/−^ mice may be attributed to decreased IgG1 titers.

The frequency of p-STAT3-positive cells was significantly decreased in the lungs of OVA-sensitized TLR2^−/−^ mice compared with OVA-sensitized wild-type mice. However, NF-κB was not activated following OVA sensitization of either TLR2^−/−^ or wild-type mice. Consistent with the work of Bieneman et al. [18],, NF-κB was not involved in the response of TLR2^−/−^ mice to OVA sensitization, nor did OVA activate NF-κB in wild-type mice. STAT3 can be activated by IL21 in vitro and can directly bind the IL21 promoter and induce IL21 expression in an autocrine positive feedback loop [37]. However, NF-κB played a role in OVA sensitization and elicited inflammation as described in our previous report [20]. Thus, clearly a difference exists between OVA sensitization and OVA sensitization that elicits inflammation. Thus, decreased STAT3 levels resulted in lower titers of OVA-specific IgG1 in OVA-sensitized TLR2^−/−^ mice. The results of our study showed that TLR2-Stat3/IL21 pathways had opposite effects on IgG1 and IgE titers following OVA sensitization.

One question was evident from these data: why did IL4, IL13 almost disappear in TLR2^−/−^ mice, but not IL5? IL4, IL13 and IL5 are produced by Th2 [38] and mast cells [39]. IL-4 is an eosinophil chemotactic factor that promotes the infiltration of airway eosinophils, and can also promote synthesis of IgE, endothelial cell adhesion factor-1, mast cell growth and goblet cell metaplasia in B cells. As a self-secreting growth factor of T cells, IL4 can promote the differentiation of naive T cells into Th2 cells, thereby inducing allergic inflammation [40–42]. IL-13 is mainly produced by activated Th2 cells, and can have synergistic effects with IL-4 to promote IgE synthesis by B cells, to activate macrophages, mast cells and eosinophils, and to inhibit eosinophil apoptosis, which can regulate goblet cells. Hyperplasia and increased mucus secretion promote the transformation of airway fibroblasts into fibroblasts, leading to collagen deposition; this can promote airway smooth muscle contraction and cause airway hyperresponsiveness [43]. IL5 is also a Th2 cytokine that induces activation, migration, proliferation and differentiation of eosinophils and inhibits apoptosis. Together, these data suggested other types of immunocytes mediate the disappearance of IL4 and IL13 in TLR2^−/−^ mice. Drake et al. [44] found that levels of IL4 and IL13 were significantly lower in the lungs of B-cell-deficient JH^−/−^ mice treated with OAAH (OVA, *Alternaria*, *Aspergillus*, and house dust mite) than in OAAH-treated wild-type mice (*p* < 0.05). Levels of IL5 were less affected than levels of IL4 or IL13. This was not due to suppression of B cell development in TLR2^−/−^ mice. On the contrary, B cell maturation is inhibited by TLR2 agonists [45]. Three studies reported that TLR2 played a role in proliferation, activation and differentiation of resting B cells [46–48]. Perhaps, TLR2 knockout inhibits B cell activation and secretion of IL4 and IL13. Takeda et al. found that eosinophil-deficient mice had increased levels of IL4, IL5, and IL13 in bronchoalveolar lavage fluid after 11 OVA challenges [49]. Thus, eosinophils were not responsible for the phenotype of TLR2^−/−^ mice. In the absence of CD11c + dendritic cells (DC), endogenous or adoptively transferred CD4+ Th2 cells did not produce IL4, IL5, and IL13 in response to OVA aerosols [50]. Thus, DCs were not likely to mediate the phenotypes of TLR2^−/−^ mice. Song et al. applied 2-chloroadenosine to deplete alveolar macrophages and found reduced levels of IL4, IL5, and IL13 in response to OVA stimulation [51]. Inhibition of early airway neutrophilia did not affect IL4, IL5, and IL13 levels [52]. The detailed mechanisms underlying these phenomena need to be explored further.

Here, Iε-Cε transcripts could not be detected in either group irrespective of OVA sensitization. Unlike for other Ig isotypes, IgE isotype switching and IgE-producing B cell expansion occur in the draining lymph node after immunization, not in the spleen [53]. Germline, post-recombination and mature transcripts could not be examined in lymph nodes of TLR2^−/−^ mice treated with IL21. This should be examined in future studies.

## Conclusions

In summary, IL4, IL13 and IL21 almost disappeared in OVA-sensitized TLR2^−/−^ mice compared with wild-type mice. However, IL21 was the most important cytokine responsible for Ig class switching in response to OVA sensitization The transcription factor regulating Ig class switching and production in OVA-sensitized TLR2^−/−^ mice was STAT3, not NF-κB. These finding may suggest potential targets for the prevention and treatment of allergic diseases.

## Data Availability

Data sharing not applicable to this article as no datasets were generated or analysed during the current study.

## Abbreviations

TLR: Toll-like receptor
Ig: Immunoglobulin
OVA: Ovalbumin
IL: Interleukin
STAT: Signal transducer and activator of transcription
NF: Nuclear factor
CPT: Cryptotanshinone
PBS: Phosphate-buffered saline
CSR: Class switch recombination
p: phosphorylated
ERK: Extracellular signal-regulated kinase
NS: Normal saline
